# Cyanobacterial flocculation as a defence against bacterial predation

**DOI:** 10.1093/ismejo/wrag169

**Published:** 2026-06-27

**Authors:** Shylaja N Mohandass, Alice C Z Collins, Fabian D Conradi, Luke P Allsopp, Conrad W Mullineaux

**Affiliations:** School of Biological and Behavioural Sciences, Queen Mary University of London, London E1 4NS, United Kingdom; School of Biological Sciences, University of Bristol, 24 Tyndall Avenue, Bristol BS8 1TQ, United Kingdom; National Heart and Lung Institute, Imperial College London, London SW3 6LR, United Kingdom; Centre for Bacterial Resistance Biology, Imperial College London, London SW7 2AZ, United Kingdom; School of Biological and Behavioural Sciences, Queen Mary University of London, London E1 4NS, United Kingdom; Institut für Tier-und Umwelthygiene, Freie Universität Berlin, Robert-von-Ostertag-Str. 8, 14163 Berlin, Germany; National Heart and Lung Institute, Imperial College London, London SW3 6LR, United Kingdom; Centre for Bacterial Resistance Biology, Imperial College London, London SW7 2AZ, United Kingdom; School of Biological and Behavioural Sciences, Queen Mary University of London, London E1 4NS, United Kingdom

**Keywords:** Cyanobacteria, flocculation, predation, *Pseudomonas aeruginosa*, *Synechocystis* sp. PCC 6803, type VI secretion system

## Abstract

Many cyanobacteria are capable of flocculation: the formation of floating linked assemblages of many thousands of cells. Flocculation is a highly regulated process requiring both type IV pilus activity and extracellular polysaccharide production. Under laboratory conditions, flocculation often slows culture growth, and its physiological advantages remain unclear. Here, we determine whether flocculation can serve as a method of defence against bacterial predation. Using the unicellular cyanobacterium *Synechocystis* sp. PCC 6803, we show that flocculation is strongly triggered by exposure to live cells of ‘foreign’ bacteria, including the ubiquitous opportunistic pathogen *Pseudomonas aeruginosa*, whose habitat overlaps with that of *Synechocystis*. The established *P. aeruginosa* virulence arsenal includes bacterial warfare systems for competition for resources and acquisition of nutrients by way of interbacterial competition. Here, we establish the use of these strategies for direct predation, via the use of type VI secretion systems. Comparisons of *P. aeruginosa* co-cultures with either wild-type *Synechocystis* or a nonflocculating mutant revealed that *Synechocystis* flocculation minimises both growth of *P. aeruginosa* and cell lysis of *Synechocystis*. This in turn reduces the impact of *P. aeruginosa* on *Synechocystis* growth by mechanistically limiting the photosynthetic products that *P. aeruginosa* can access. From these data, we propose that the type VI secretion system of *P. aeruginosa* can be used for predation and that a major function of cyanobacterial flocculation is for defence against microbial predation.

## Graphical Abstract

**Graphical Abstract:**
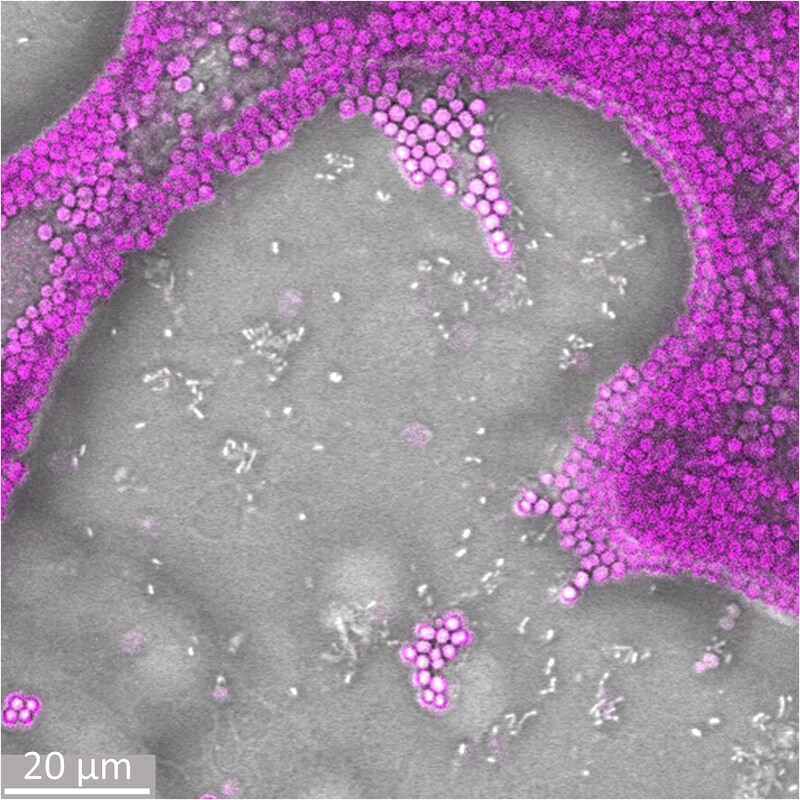

## Introduction

Cyanobacteria are globally important as primary producers and are found in a wide variety of marine, freshwater, and terrestrial habitats [1]. Much research on cyanobacteria has focused on their oxygenic photosynthesis and associated autotrophic metabolism; however, cyanobacteria have other fascinating aspects to their biology. Filamentous cyanobacteria provide examples of sophisticated prokaryotic multicellularity [2], and unicellular cyanobacteria are capable of complex and co-operative behaviour [3]. For example, the unicellular freshwater cyanobacterium *Synechocystis* sp. PCC 6803 (hereafter *Synechocystis*) can under suitable conditions form large floating assemblages or flocs, which can contain thousands of cells [3–7]. *Synechocystis* floc formation requires both the type IV pilus (T4P) apparatus [6, 7] and the production of a specific sulfated exopolysaccharide termed synechan [4]. Floc formation is tightly regulated and promoted by environmental factors including nutrient limitation [6] and blue light acting via the cyanobacteriochrome photoreceptor Cph2 [7].

*Synechocystis* cells in the centre of dense flocs show signs of nutrient stress [7] and comparison of wild-type (WT) *Synechocystis* to a nonflocculating mutant shows that flocculation slows growth under standard laboratory conditions [7]. This hints at some unknown benefit of flocculation. One possibility is that flocculation promotes self-shading and reduces exposure to excessive or harmful light. In the thermophilic cyanobacterium *Thermosynechococcus vulcanus*, flocculation is induced by low temperatures and blue light [8] to promote self-shading. This requires production of extracellular cellulose stimulated by a network of photoreceptors acting through the intracellular second messenger cyclic di-GMP [9, 10]. In *Synechocystis*, flocculation has been suggested to benefit cells by trapping gas bubbles for flotation, potentially allowing cells to find a more favourable level in the water column [4]. Additionally, flocculation has been proposed to promote formation of mutualistic bacterial communities. *Synechocystis* was previously shown to form more durable biofilms in the presence of a heterotrophic partner, *Pseudomonas taiwanensis* [11], suggesting a mutualistic relationship in which *P. taiwanensis* aerobic metabolism prevents the excessive build-up of oxygen in the biofilm [11], which may be extrapolated to floating flocs [3].

Here, we test the possibility that cyanobacterial flocculation serves as a defence mechanism against predation. We selected the opportunistic pathogen *Pseudomonas aeruginosa* as a model bacterial predator, as it actively engages in bacterial warfare [12–14]. *Pseudomonas aeruginosa* is found in soils and freshwater bodies [15], and therefore, its habitat overlaps with that of *Synechocystis*, which is found in fresh and brackish water [16]. *Pseudomonas aeruginosa* has also been used as a model for the interaction of heterotrophic bacteria with marine particles [17].

*Pseudomonas aeruginosa* lyses competitor cells using an arsenal of weapons, including contact-independent cell lysis molecules such as bacteriocins, pyocins, and porins [12, 18], and contact-dependent systems such as the type VI secretion system (T6SS) [12–14]. Bacterial warfare studies typically focus on heterotrophic species and are considered a competition for resources [13, 14, 19]. However, antibacterial weapons could also be employed for direct predation. As *P. aeruginosa* is dependent on organic molecules for survival, predation on *Synechocystis* may be significant in co-culture in a mineral medium in which *Synechocystis* grows photoautotrophically but which lacks organic molecules that could directly feed *P. aeruginosa*. Here, we studied how *P. aeruginosa* interacts with *Synechocystis* when co-cultured in mineral BG11 medium [20]. We show that successful predation on *Synechocystis* cells by *P. aeruginosa* is predominantly accredited to the action of the T6SSs. In response, *Synechocystis* employs flocculation as a defence mechanism against *P. aeruginosa* predation.

## Materials and methods

### Bacterial strains and growth conditions

*Synechocystis* strains used were the motile PCC-M substrain of *Synechocystis* sp PCC 6803 [21] and the Δ*hfq* null mutant constructed in the same background [22]. Hfq is known as an RNA-binding protein in other bacteria, but in *Synechocystis*, it interacts with the T4P machinery [23] and the major functional effect of its deletion is to abolish motility [22] and flocculation [7]. Sy*nechocystis* starter cultures were grown in BG11 medium [20] supplemented with 2-{[1,3-Dihydroxy-2-(hydroxymethyl)propan-2-yl]amino}ethane-1-sulfoniacid (TES) buffer (pH 8.2) at 30°C in plastic tissue culture flasks (Sarstedt), under continuous white light illumination (~15 μmol photons m^−2^ s^−1^) with shaking (120 rpm). Cultures were also maintained on BG11 plates containing 1.5% (w/v) Bacto-agar (VWR) supplemented with TES buffer (pH 8.2) and 0.3% (w/v) sodium thiosulfate. TES is a standard addition to BG11 agar plates that we also used for starter cultures but omitted from experimental assays to keep the medium free of organic compounds. Plates and starter cultures were maintained in lower light than for the experimental assays.

The heterotrophic bacteria (*Escherichia coli* Top10 from Thermo-Fisher Scientific and *P. aeruginosa* strains) were cultured in LB liquid medium and on LB agar plates (Thermo-Fisher Scientific) at 37°C with appropriate antibiotics. All *P. aeruginosa* strains were in the PA14 background [24] including the mutant *prtN::tn* [25], which lacks the positive regulator required for pyocin production [26], and the newly constructed H123^−^, which lacks all three of the *P. aeruginosa* T6SS (H1-, H2-, and H3-T6SS) [12].

### Construction of the PA H123^−^ mutant

Genomic, PCR, and plasmid vector DNA was purified using Qiagen DNeasy Blood and Tissue, QIAquick PCR Purifications kit and QIAprep Spin Miniprep kits, respectively. DNA fragments were amplified with either KOD Hot Start DNA Polymerase (Merck) or standard Taq polymerase (NEB) as described by the manufacturer with the inclusion of Betaine (Sigma) or DMSO (Sigma). Restriction endonucleases and ligase were used according to the manufacturer’s specifications (NEB). All constructs were DNA sequenced and confirmed to be correct before use by Eurofins Genomics. Plasmids were introduced into *E. coli* strains by heat shock transformation and to *P. aeruginosa* strains by conjugation.

*Pseudomonas aeruginosa* PA14 mutagenesis used derivatives of the pKNG101 plasmid, which has an origin of replication that works in *E. coli* but not in *P. aeruginosa*, plus a streptomycin resistance gene for selection and a *sacB* gene for counterselection on sucrose. The plasmid is engineered to contain homology regions that will guide integration into the *P. aeruginosa* chromosome at desired sites, transferred to *P. aeruginosa* via conjugation and transformants where the plasmid has integrated into the chromosome are selected via streptomycin resistance. Integration is confirmed by PCR, and strains are then switched to growth without streptomycin. Counter selection on sucrose then selects for recombinants that have lost the integrated pKNG101 backbone. Strain PA14 H123^−^ was engineered in this study by restoring the WT copy of *rsmA* in the PA14*rsmA* H123^−^ [27] background [*rsmA*, H1-T6SS (*tagQ1* -*vgrG1b*), H2-T6SS (*tssA2-clpV2*), and H3-T6SS (*tssB3* -*clpV3*)]. The pKNG101 derivative used, pKNGrsmA restore, was generated by amplifying the WT copy of *rsmA* using primers P1 and P2 (Table S1) and cloning this into pKNG101 using ApaI and SmaI [27].

### Flocculation assays

Freshly cultured *Synechocystis* cells were pelleted by centrifugation at 4000 × *g* for 5 min and resuspended in fresh BG11 medium (without TES buffer, as this is a potential organic food source for heterotrophs) to an OD_750_ of 0.5 (Jenway 6300 spectrophotometer). Cell density was ~2.8 × 10^8^ cells ml^−1^ as assessed by counting in a haemocytometer (Neubauer counting chamber). The resuspended *Synechocystis* cultures were transferred into Corning Costar TC-treated 6-well plates (Sigma-Aldrich) with 5 ml of culture per well. Where appropriate for co-culture assays, overnight cultures of the heterotrophic bacteria *(P. aeruginosa*, *E. coli*) were standardized to OD_600_ of 1.5 (equating to a cell density of ~9 × 10^8^ ml^−1^ for *P. aeruginosa*) and a 50 μl aliquot was added to the 5 ml of *Synechocystis* culture in each well. For flocculation assays with additional *Synechocystis* cells, a *Synechocystis* culture was concentrated to ~9 × 10^8^ cells ml^−1^ and a 50 μl aliquot added to the well. The 6-well plates were then sealed and incubated at 30°C for 48 h with white light illumination at 30 μmol photons m^−2^ s^−1^ with shaking at 75 rpm (16-mm orbit diameter/2-mm stroke in an SI50 orbital incubator; Stuart Scientific, UK). All assays were performed in triplicate. After 48 h growth, the 6-well plates were removed from the incubator, placed on a light box (Medalight LP-300 N cold cathode fluorescent lamp), and imaged using an Olympus OM-D (E-M1 Mark II) camera vertically positioned on a stand (Kaiser 205361) at a height of 40 cm. All images were taken with standard specifications (ISO 200, shutter speed 40, F5.6, default white balance). Olympus Capture software was used for positioning and focusing. Images were analysed with Image J [28] and numerical aggregation values were calculated from the normalized standard deviation of the images as in [7]. This aggregation score provides a quantitative metric of the inhomogeneity of the distribution of cells in the well, although it is unlikely to scale linearly with the density of cells in the flocs.

### Growth measurements

*Synechocystis* and *P. aeruginosa* cell densities in co-culture were assessed at the start of the experiment and after 48 h. The contents of the wells were transferred to 50 ml tubes (Sarstedt) and vigorously shaken and vortexed to disperse the flocs and ensure even distribution of the cells. *Pseudomonas aeruginosa* cells in the co-culture were then quantified by colony-forming unit (CFU) assays. Co-culture samples were serially diluted 10-fold in BG11 medium (without TES buffer), and 10 μl aliquots were spotted onto *Pseudomonas* isolation agar (Millipore, Sigma-Aldrich) followed by incubation overnight at 35°C and colony counting.

Growth of *Synechocystis* in co-culture was assessed by measuring chlorophyll *a* as a proxy for cell density, due to the difficulty of direct counting of aggregated cells, challenges in distinguishing live and dead cells, and complications with CFU assays arising from the slow appearance of colonies and the potential for further attack by *P. aeruginosa* in co-cultures. Chlorophyll content per cell could potentially be variable, but we saw no indication of differences in chlorophyll content in healthy cells as judged from our chlorophyll fluorescence micrographs. Chlorophyll in 1 ml aliquots was extracted with methanol and the concentration calculated from absorbance at 665 nm as previously described [29].

### Extracts from cultures

A variety of extracts from cell cultures were tested for their influence on *Synechocystis* flocculation. In all these cases, flocculation was assessed after 24–28 h. Spent *P. aeruginosa* medium was obtained by centrifuging aliquots of overnight grown *P. aeruginosa* cultures (OD_600_ 1.5) at 10 000 × *g* for 10 min. The supernatant was then filter-sterilized using a 0.2 μm syringe filter (Fisher Scientific). Fifty microlitres of filtered spent medium was added to the well for the assay. For the spent medium from co-cultures, *P. aeruginosa*–*Synechocystis* co-cultures were grown as described above for flocculation assays. Cells were then pelleted by centrifugation, and the supernatant was filtered and added to flocculation assays as for the spent *P. aeruginosa* medium.

Extracellular polymeric substances (EPSs) were extracted by ethanol precipitation according to [30, 31]. Supernatants from 100 ml of freshly grown *P. aeruginosa* cultures were mixed with 95% ethanol in a 1:3 ratio (v/v) and incubated at 4°C overnight to precipitate EPS. The EPS was separated by centrifuging the mixture at 4000 × *g* for 20 min followed by incubation of the pellet at 37°C to evaporate the solvent. The pellet was resuspended in double-distilled water to 0.4% w/v by vigorous mixing until it completely dissolved. The solution was then passed through a 0.45 μm cellulose acetate membrane (Thermo-Fisher Scientific) to remove any remaining cells. Subsequently, a volume of 50 μl of the extracted EPS was added to the flocculation assay.

*Synechocystis* cell lysates were prepared by mixing 350 μl of healthy *Synechocystis* cells at an OD_750_ of 0.5 with 150–212 μm glass beads (Sigma) at a 1:1 ratio in an Eppendorf tube. The mixture was vortexed for 10 cycles using a Disruptor Genie (Scientific Industries), with each cycle consisting of 1 min of disruption followed by a 1-min pause at 4°C. The lysate was then centrifuged at 13 000 × *g* for 10 min, and 50 μl of the supernatant was added to a flocculation assay.

Heat-killed but intact *P. aeruginosa* cells were prepared according to [32] with some modification. Overnight cultures at an OD_600_ of 1.5 were centrifuged at 12 000 × *g* for 10 min, and the pellet was resuspended in the same volume of BG11 medium (without TES buffer) followed by incubation at 92°C for 2 h. Fifty microlitres of the heat-killed culture was added to a flocculation assay.

### Preparation of samples for microscopic imaging

Floc samples from the co-culture assay were carefully removed using 1 ml Gilson pipettes with the end of the pipette tip cut off to enlarge the hole, and gently pressed between two 24 × 60 mm microscope coverslips. The edges were sealed to keep the flocs stationary during imaging. EPS (β-polysaccharide) in the flocs was visualized using calcofluor white staining (Sigma-Aldrich). Flocs were gently transferred to a Nunc Lab-Tek 2-well chamber slide using blunt pipette tips, followed by addition of calcofluor white (300 mg L^−1^, 100 μl) [33]. The slides were then incubated in the dark for 30 min at 30°C. After incubation, the flocs were carefully washed with phosphate-buffered saline (PBS) buffer and placed between microscope coverslips as before.

### Confocal imaging and image analysis

All imaging was with a Leica Stellaris 8 confocal laser scanning microscope equipped with a 63× oil immersion objective (numerical aperture 1.4). Chlorophyll fluorescence from *Synechocystis* cells was imaged with excitation from a 405 nm diode laser and emission at 670–720 nm. *Pseudomonas aeruginosa* was visualized in brightfield mode. Calcofluor white fluorescence was excited at 405 nm and detected at 420–490 nm. Images were recorded in 8-bit, 512 × 512 pixel format with 8× line averaging at 400 Hz scan speed and visualized with Leica LAS AF software. Widefield images were obtained in tiling mode, combining sets of images recorded as before except without line averaging. Each tile in the grid was autofocused before tile scanning.

*Synechocystis* cell area and perimeter analysis were performed in ImageJ using the chlorophyll fluorescence images. Cell boundaries were determined by thresholding and individual cells in clear view were selected. Cells undergoing division were handled manually.

## Results

### Flocculation of *Synechocystis* is triggered by exposure to foreign bacteria

**Figure 1:**
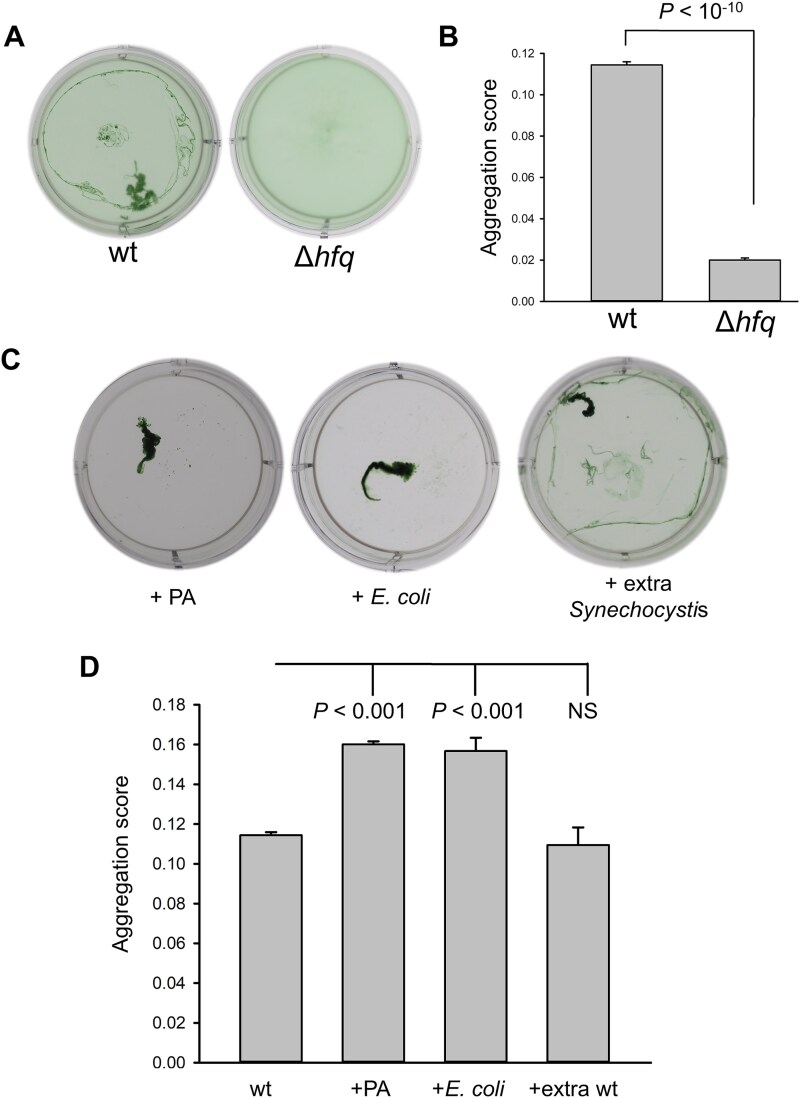
*Synechocystis* flocculation is triggered by exposure to foreign bacteria. (A) Representative flocculation assays for pure cultures of *Synechocystis* wild type and Δ*hfq*. (B) Mean aggregation values for pure cultures of wild type and Δ*hfq*. Error bars represent the standard error of the mean (SEM) from biological replicates (*n* = 6). Normality was assessed using the Shapiro–Wilk test. Statistical significance between the indicated groups was determined using a two-tailed unpaired Student’s *t-*test. (C) Representative flocculation assays for *Synechocystis* wild type co-cultured with *P. aeruginosa* PA14, Top10 *E. coli,* or with the addition of an equivalent amount of extra *Synechocystis.* (D) Mean aggregation values for flocculation assays of the co-cultures. All measurements are means from three biological replicates. Error bars represent SEM from biological replicates [*n* = 6 for three samples (wt, +PA, + extra wt); *n* = 3 for one sample (+*E. coli*)]. Normality was assessed using the Shapiro–Wilk test. Statistical significance was determined using one-way ANOVA followed by Dunnett’s multiple comparisons test comparing each group to wild-type *Synechocystis*.

**Figure 2:**
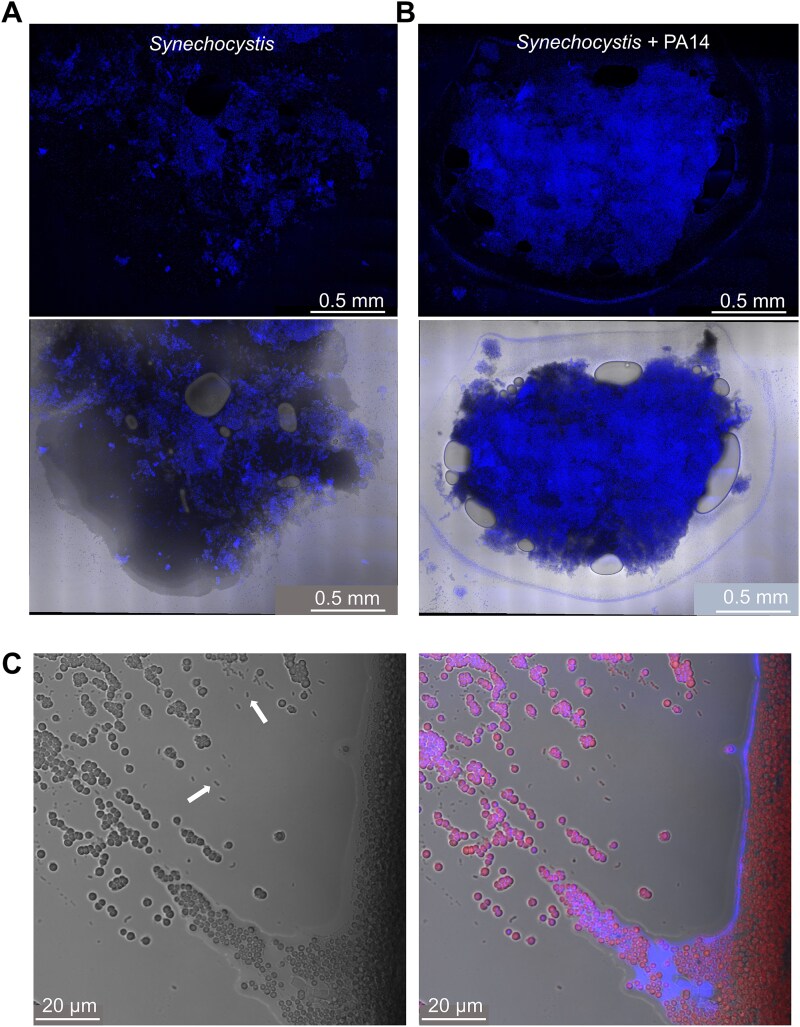
Dense *Synechocystis* flocs are associated with high concentrations of extracellular polysaccharide. (A, B) Wide-angle microscopic images of floc samples showing Calcofluor white fluorescence (in blue) merged with brightfield (grayscale) in the lower images. (A) Floc from a *Synechocystis* pure culture. (B) Floc from a *Synechocystis* co-culture with *P. aeruginosa* PA14. (C) Higher-magnification image of the edge of a floc from a *Synechocystis*:*P. aeruginosa* co-culture. Brightfield image on the left: *P. aeruginosa* cells are visible as small rods, with examples highlighted by the arrows. On the right is a merged image with brightfield plus chlorophyll fluorescence (red) and Calcofluor white fluorescence (blue).

*Synechocystis* has evolved in laboratory cultures into a range of substrains with different phenotypic characteristics [34]. Here, we used the PCC-M substrain [21], which is highly motile on surfaces and readily flocculates in planktonic cultures [7]. We induced and quantified flocculation in *Synechocystis* using a method previously outlined [7], in which planktonic cultures are grown using BG11 medium in 6-well plates with gentle shaking, where flocculation was recorded after 48 h. BG11 medium supports photosynthetic growth but lacks organic carbon sources [20]. To quantify flocculation, we recorded standardized digital camera images of the wells and measured the normalized standard deviation in the image pixel values, defining a numerical aggregation score. A high standard deviation indicates an inhomogeneous cellular distribution within the well and therefore a high level of flocculation or aggregation of the cells [7]. Our control, a Δ*hfq* mutant, lacks cell appendages [22] and does not flocculate [7]. As expected, aggregation scores corroborate WT flocculation under our conditions, whilst Δ*hfq* cells remained evenly dispersed and consequently give much lower aggregation values (Fig. 1A and B). To test the idea that *Synechocystis* flocculation could be triggered as a defence mechanism against foreign bacteria, we grew co-cultures in the presence of the potentially aggressive *P. aeruginosa* PA14 [24] and found that this induced the formation of much denser flocs (Fig. 1C). To test whether this might be a specific response to this species, or to predatory attack, we tried a similar experiment with the nonpathogenic laboratory strain *E. coli* Top10, which is widely used as a benign prey strain in intermicrobial competition assays [27], and found that both species induced similar very dense flocs (Fig. 1C), which was reflected in the change in aggregation scores observed when compared to WT *Synechocystis* monocultures (Fig. 1D). To check if the enhanced flocculation was simply a cell density–driven response, we supplemented the wells with a similar number of extra *Synechocystis* cells; however, this had no effect (Fig. 1C and D). Next, we used calcofluor white staining [35] to investigate polysaccharide production in *Synechocystis* flocs. Flocs from *Synechocystis* monoculture generate some extracellular polysaccharide but have a relatively open structure (Fig. 2A). However, exposure to *P. aeruginosa* induced increased production of extracellular polysaccharide (Fig. 2B) that appeared to form a protective barrier at the floc edge (Fig. 2C).

### *Synechocystis* flocculation is triggered by exposure to live or heat-killed *P. aeruginosa* cells

**Figure 3:**
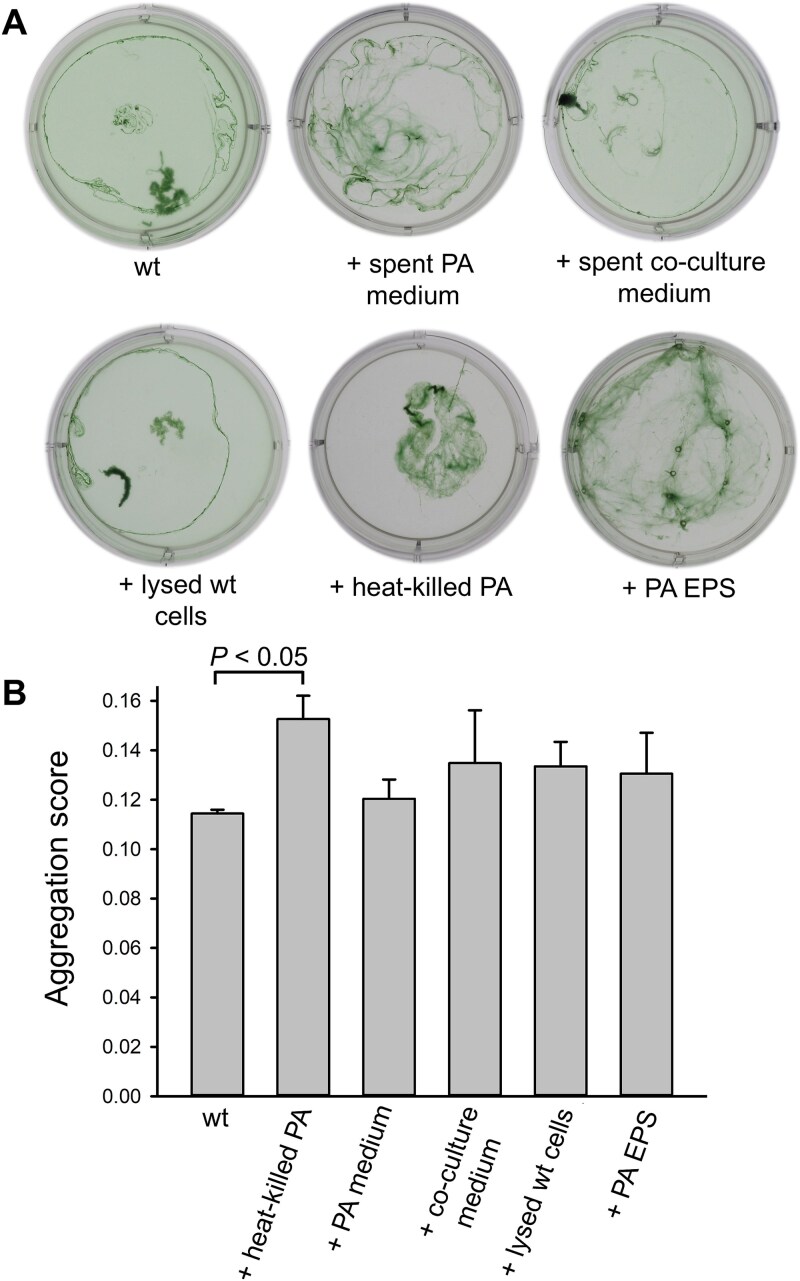
Effects of culture extracts on *Synechocystis* flocculation. (A) Representative flocculation assays for *Synechocystis* wild type incubated with heat-killed *P. aeruginosa* cells, filtered medium from *P. aeruginosa* cultures and *P. aeruginosa*:*Synechocystis* co-cultures, mechanically lysed *Synechocystis* cells, and *P. aeruginosa* EPS extracts. (B) Mean aggregation values from these conditions. Error bars represent the standard error of the mean (SEM) from biological replicates (*n* = 6 for wt; *n* = 3 for the other conditions). Statistical significance was determined using the Mann–Whitney *U* test.

**Figure 4:**
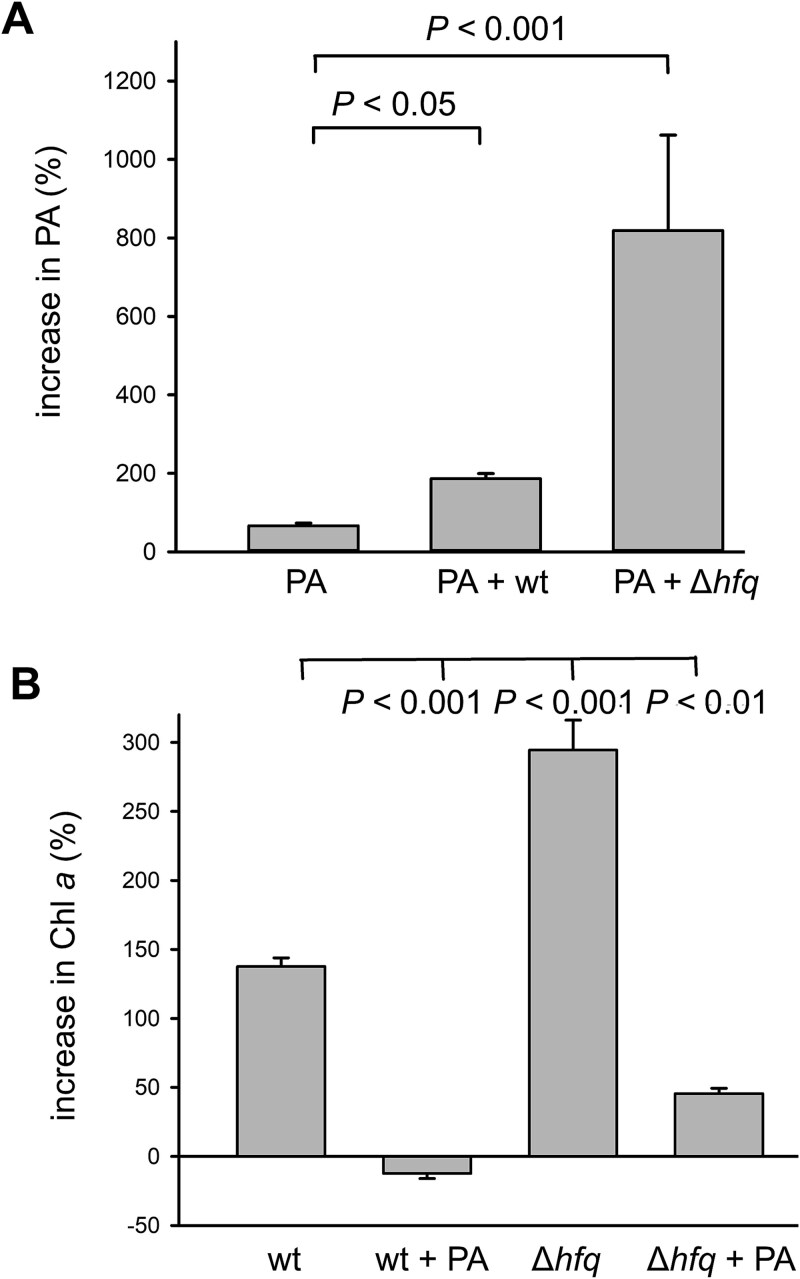
Growth of *Synechocystis* and *P. aeruginosa* in co-culture for 48 h. (A) Growth of *P. aeruginosa* (CFU ± SEM) in BG11 medium in pure culture or together with *Synechocystis* wild type or Δ*hfq.* (B) Growth of *Synechocystis* strains in pure culture or together with *P. aeruginosa*, assessed from chlorophyll concentration ± SEM (*n* = 3). Raw data are provided in Tables S2 and S3. Statistical differences were assessed by one-way ANOVA (following log transformation in the case of Fig. 4A) followed by post hoc analysis using Dunnett’s test, using wt *Synechocystis* or Δ*hfq* as controls.

To explore potential factors responsible for triggering denser *Synechocystis* flocculation, we tested the effects of filtered media from *P. aeruginosa* monocultures and *Synechocystis*: *P. aeruginosa* co-cultures upon flocculation after 48 h. The media were filtered with a 0.2 μm syringe filter, and hence, they will contain small molecules and proteins excreted into the medium but not cells or larger extracellular polymers. We observed no obvious effects (Fig. 3). Since kin cell lysis can serve as a bacterial danger signal [36], we next added mechanically broken *Synechocystis* cells, again with no obvious effect (Fig. 3). Similarly, an extract of PA14 EPS failed to induce additional flocculation (Fig. 3). We then explored the effect of addition of heat-killed PA14 cells, which did significantly elevate the aggregation scores with a visible effect (Fig. 3), although these flocs appeared less compact and dense than those induced by live PA14 cells (Figs 1 and 3). We cannot exclude that this effect may have been due to some released PA14 cell debris in the preparation, and we did not test spent *E. coli* media or cell extracts. However, taken together, the results suggest that *Synechocystis* flocculation is induced by contact with foreign bacterial cell surfaces. The greater response to live *P. aeruginosa* cells (Fig. 1) compared to heat-killed cells (Fig. 3) may be due to cell proliferation during the 48 h incubation (Fig. 4) and/or be due to *P. aeruginosa* specifically targeting *Synechocystis* through an active mechanism [37].

### Impact of *Synechocystis* flocculation on growth of *P. aeruginosa* in co-culture

We quantified growth of *Synechocystis* and *P. aeruginosa* over 48 h in BG11 medium. *Pseudomonas aeruginosa* growth was assessed by counting CFUs from the co-cultures on *Pseudomonas* isolation agar, on which *P. aeruginosa* grows rapidly but *Synechocystis* does not. As expected, *P. aeruginosa* cell growth in monoculture was minimal, as BG11 medium lacks organic carbon sources (Fig. 4A). To assess the additional impact of *Synechocystis* flocculation on growth, we compared co-cultures using either WT *Synechocystis* or the non-flocculating Δ*hfq* mutant [7]. Here, a significant increase in *P. aeruginosa* growth occurred in WT *Synechocystis* co-culture, but *P. aeruginosa* growth was greater in co-culture with Δ*hfq* (Fig. 4A)*.*

Comparison of the impact of *P. aeruginosa* on growth of WT *Synechocystis* vs. Δ*hfq* is complicated, as Δ*hfq* monocultures grow faster than wild type under our culture conditions (Fig. 4B), likely because flocculation reduces the efficiency of light absorption and mineral supply to cells in the centre of flocs [7]. Nevertheless, chlorophyll concentration at 48 h shows that the presence of *P. aeruginosa* greatly impacts *Synechocystis* (Fig. 4B), and the population deficit is higher for Δ*hfq* than for WT (Fig. 4B; Table S3). The deficit in chlorophyll concentration induced by exposure to *P. aeruginosa* is 3.87 ± 0.38 μM for WT and 5.24 ± 0.51 μM for Δ*hfq* (mean ± SD; *P* = .02 from unpaired Student’s *t-*test). This implies that more Δ*hfq* cells than WT cells are being killed by *P. aeruginosa* and is consistent with the faster proliferation of *P. aeruginosa* in co-culture with Δ*hfq* (Fig. 4A).

**Figure 5:**
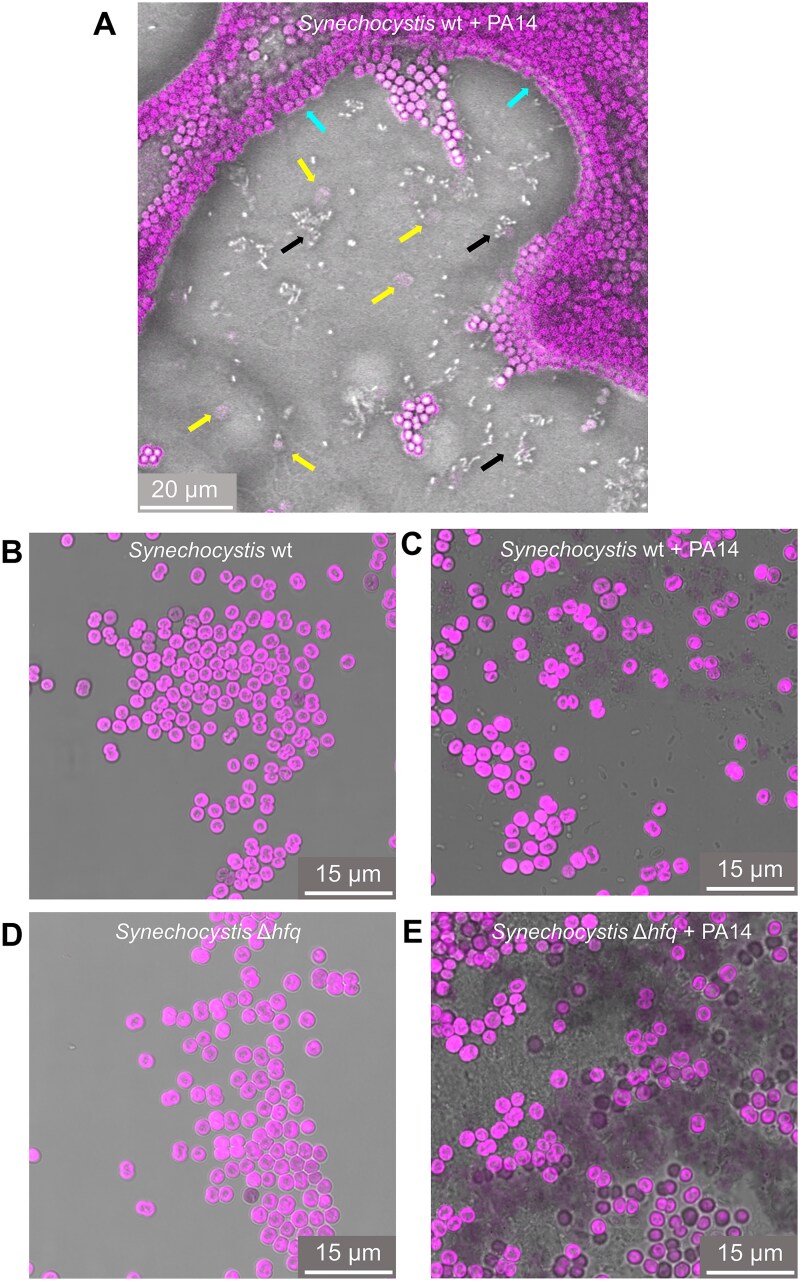
Addition of *P. aeruginosa* to *Synechocystis* cultures results in cell death. Chlorophyll fluorescence from *Synechocystis* cells is shown in magenta, merged with brightfield images in grayscale. (A) The edge of a *Synechocystis* floc (wild type plus *P. aeruginosa*). Note *Synechocystis* cells (e.g. cyan arrow), *P. aeruginosa* cells (e.g. black arrows), and some very pale *Synechocystis* cells that appear to have been lysed (e.g. yellow arrows). (B–E) Representative images from cultures and co-cultures. (B) *Synechocystis* wild type. (C) Wild type plus *P. aeruginosa*. (D) *Synechocystis* Δ*hfq.* (E) Δ*hfq* plus *P. aeruginosa*. Note the presence of *P. aeruginosa* cells and compromised *Synechocystis* cells with indistinct edges and minimal fluorescence in (C) and (E).

The impacts of co-culture on both *P. aeruginosa* and *Synechocystis* growth (Fig. 4B) suggest that *P. aeruginosa* may be actively predating *Synechocystis* cells for survival rather than just utilizing secreted photosynthetic products. Microscopic examination of the co-cultures (Fig. 5A, C and  E) highlighted the presence of lysed *Synechocystis* cells, which were rarely observed in pure cultures (Fig. 5B and D). A significant proportion of the *Synechocystis* cells become indistinct in brightfield and show much lower chlorophyll fluorescence in co-culture (Fig. 5C and E), indicating cell lysis and subsequent loss or degradation of photosynthetic pigments. *Pseudomonas aeruginosa* cells are observable as much smaller, nonfluorescent rods, and are often seen clustering around *Synechocystis* cells, hinting at the occurrence of a contact-dependent interaction (Fig. 5C and E; Fig. S1).

### *P. aeruginosa* uses multiple mechanisms to lyse *Synechocystis* cells

**Figure 6:**
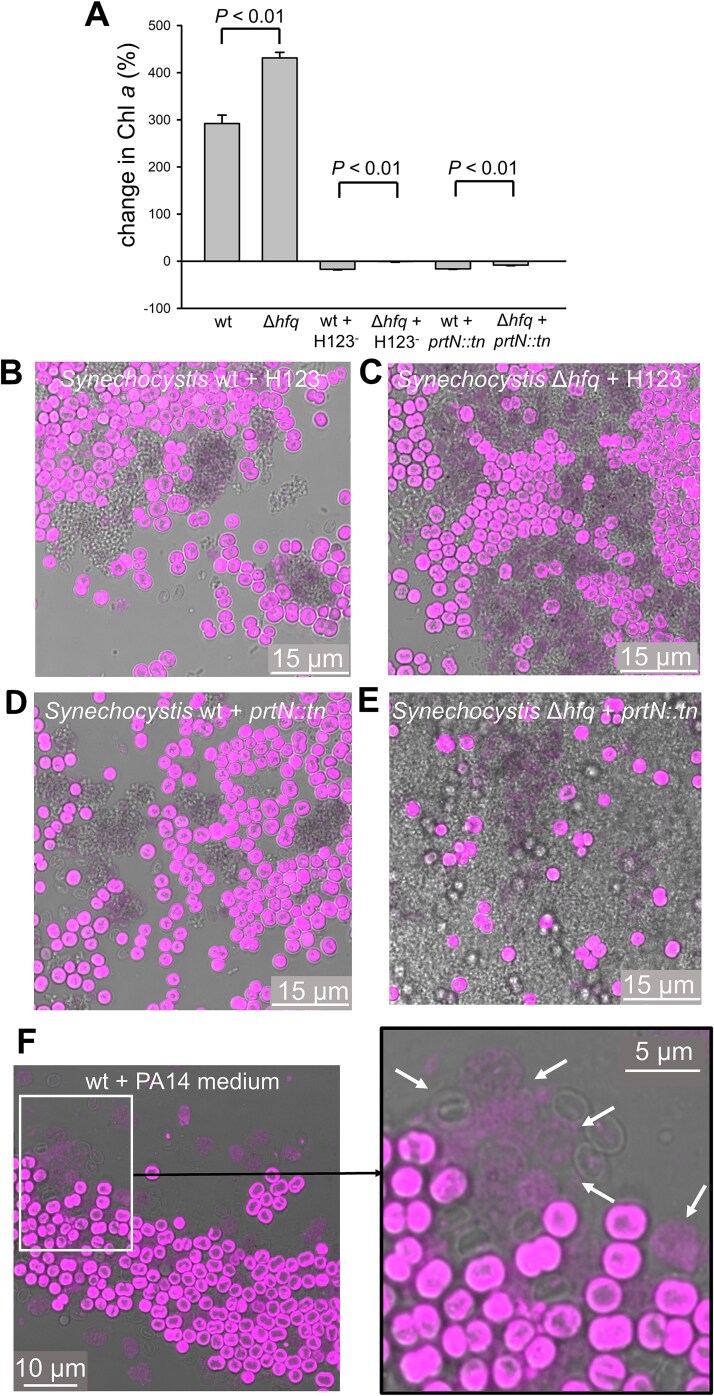
Effects of *P. aeruginosa* mutants and spent medium on *Synechocystis* strains. (A) Growth of *Synechocystis* wild type and ∆*hfq* in co-culture with *P. aeruginosa mutants*, assessed from chlorophyll concentration after 48 h. Raw data are in Table S4. Normality was assessed using the Shapiro–Wilk test. Statistical significance between the indicated groups was determined using a two-tailed unpaired Student’s *t-*test. (B–E) Fluorescence micrographs showing *Synechocystis* cell death induced by the PA14 mutants. Chlorophyll fluorescence in magenta merged with brightfield (grayscale). (B) Wild-type *Synechocystis* with PA14 H123^−^. (C) *Synechocystis* ∆*hfq* with PA14 H123^−^. (D) Wild-type *Synechocystis* with PA14 *prtN::tn*. (E) *Synechocystis* ∆*hfq* with PA14 *prtN::tn*. (F) Fluorescence micrograph, with detail, showing *Synechocystis* cells compromised by exposure to filtered *P. aeruginosa* medium. Arrows highlight *Synechocystis* cells in various stages of collapse.

To explore the mechanisms that *P. aeruginosa* uses to lyse *Synechocystis* cells, we set up co-cultures with *P. aeruginosa* PA14 mutants deficient in either contact-dependent or contact-independent antibacterial weapons. PA14 H123^−^ lacks all three of the *P. aeruginosa* T6SS [12] whilst PA14 *prtN::tn* [25] lacks the positive regulator required for pyocin production [26]. Both mutants strongly impacted *Synechocystis* growth in co-culture (Fig. 6A), and microscopic examination indicated noticeable *Synechocystis* cell death induced by both mutants (Fig. 6B–E). In line with this, we found that filtered *P. aeruginosa* culture media triggered the appearance of large numbers of ‘ghost’ *Synechocystis* cells with minimal chlorophyll fluorescence (Fig. 6F): such cells were rarely seen in healthy *Synechocystis* cultures (Fig. 5B and D) and support a cell contact–independent mechanism such as pyocins. Additionally, the pronounced effects seen with the PA14 H123^−^ mutant indicate that a contact-independent mechanism can induce *Synechocystis* cell death.

**Figure 7:**
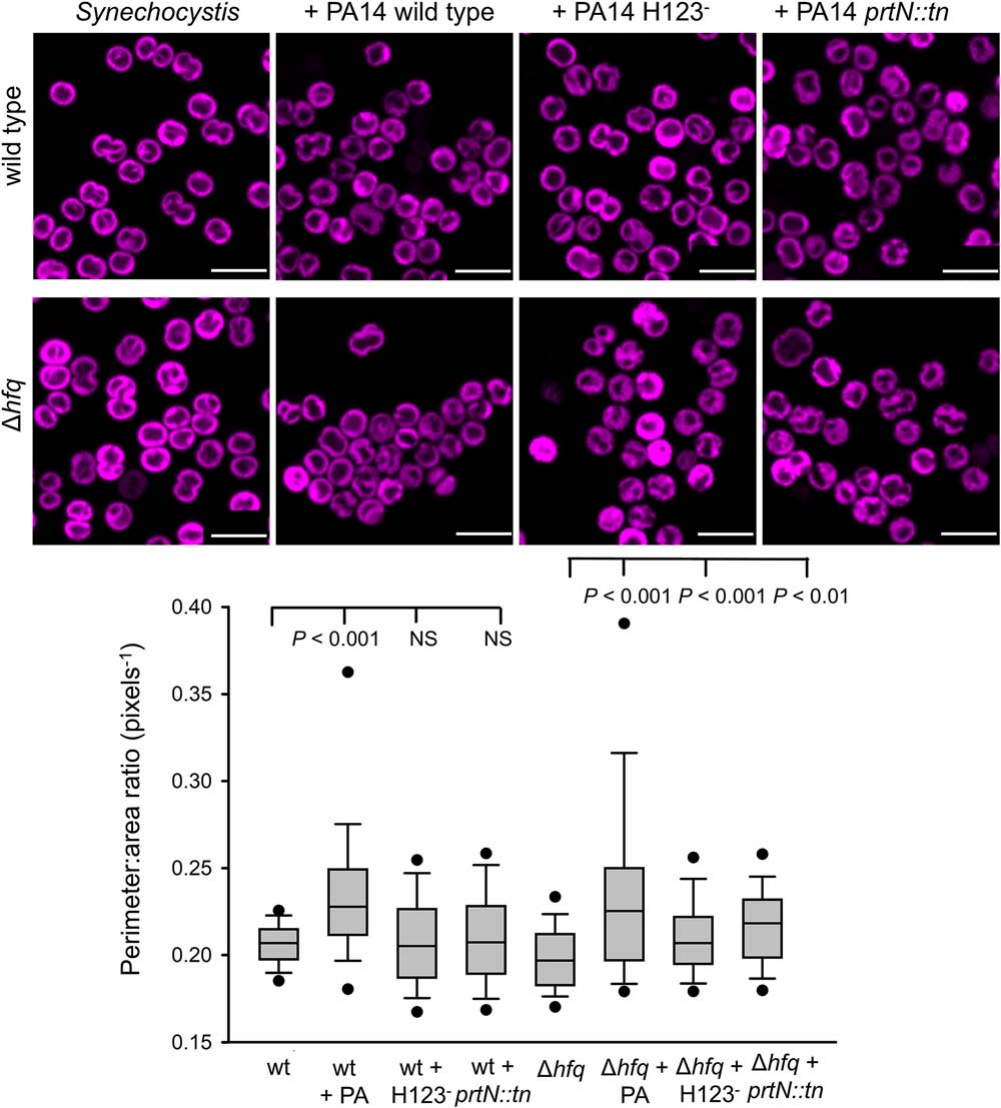
*Synechocystis* cell structure changes induced by exposure to *P. aeruginosa* wild type and mutants. Specimen chlorophyll fluorescence micrographs are shown at the top (all scale bars 5 μm) and quantitation of the cell perimeter:area ratio is shown below (*n =* 100 cells in each case). Data presented as box plots showing the median (centre line), interquartile range (box), and whiskers representing 1.5× the interquartile range. Points beyond the whiskers indicate outliers. Statistical significance was assessed using a Kruskal–Wallis test followed by Dunn’s multiple comparisons *post hoc* test. *n* = 100, comparing wt *Synechocystis* and Δ*hfq* in pure culture versus co-culture.

To comprehensively examine the effects of *P. aeruginosa* PA14 WT and mutant cells on *Synechocystis*, fluorescence micrographs of *Synechocystis* cells from the co-cultures were investigated to visualize the native chlorophyll fluorescence from the thylakoid membranes. Healthy cells are roughly spherical in shape. The thylakoid membrane layers in these cells are rather irregular, but their distal surfaces are quite smooth because they are contained within the smooth cell envelope layers. In cells from *P. aeruginosa* co-cultures, a proportion of cells had convoluted distal surfaces to their thylakoid membranes, suggesting disruption of the cell envelope. We quantified this effect by measuring the perimeter:area ratio, and found significant increases in the mean ratio for WT *Synechocystis* with WT PA14 co-culture, and for *Synechocystis* Δ*hfq* with either WT, H123^−,^ or *prtN::tn* PA14 co-culture (Fig. 7). The presence of these disrupted cells indicates direct cell attack that may be mediated in either contact-dependent or contact-independent fashion.

### Contact-dependent weapons are more effective for supporting growth of *P. aeruginosa* in co-culture

**Figure 8:**
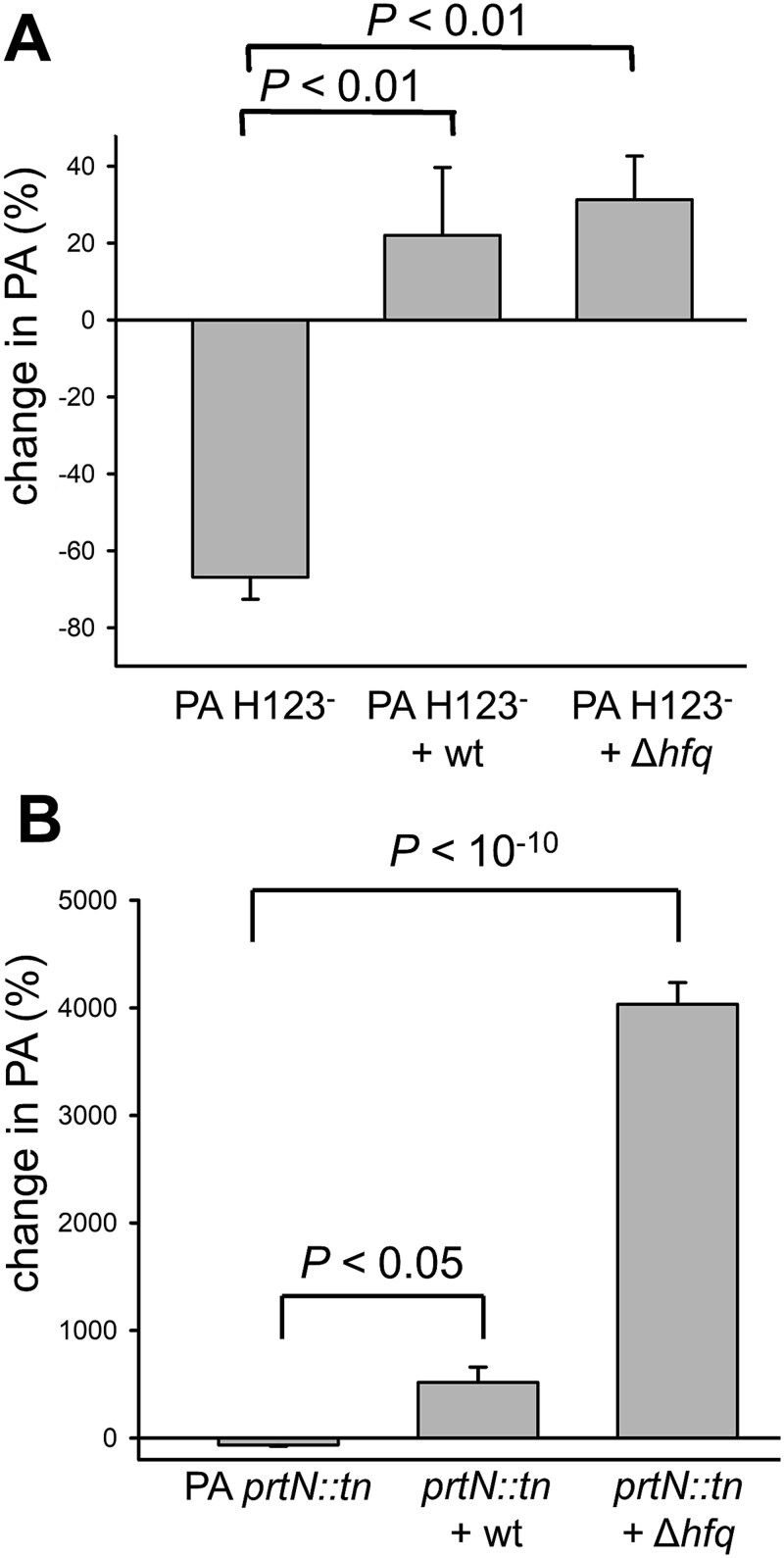
Growth of *P. aeruginosa* mutants in co-culture with *Synechocystis* strains. Growth was assessed from CFU counts after 48 h of co-culture. Data are means from three biological replicates, and error bars indicate SEM. (A) PA14 H123^−^, lacking T6SS. (B) PA14 *prtN::tn*, deficient in production of pyocins. Raw data are in Tables S5 and S6. Differences between growth of PA14 *prtN::tn* and PA14 H123^−^ are significant: *P* = .026 for comparison of the PA14 strains in co-culture with *Synechocystis* wt; *P* = .0004 for comparison of the PA14 strains in co-culture with *Synechocystis* Δ*hfq*. Normality was assessed using the Shapiro–Wilk test. Statistical significance was determined using one-way ANOVA followed by Dunnett’s multiple, comparing the *P. aeruginosa* growth in pure culture versus co-culture. Note that proliferation of PA14 H123^−^ is much slower than proliferation of PA14 *prtN::tn* in this condition.

Although the two PA14 mutants were similarly effective in killing *Synechocystis* cells (Figs 6 and 7), there were striking differences in their growth in co-culture with *Synechocystis* strains (Fig. 8). As expected, both mutants failed to grow in BG11 medium in the absence of *Synechocystis* (Fig. 8A and B). In the presence of *Synechocystis* WT or Δ*hfq*, PA14 H123^−^ showed only very modest growth after 48 h (Fig. 8A); however, PA14 *prtN::tn* proliferated strongly, especially in co-culture with *Synechocystis* Δ*hfq* (Fig. 8B). In this situation, growth of PA14 *prtN::tn* (Fig. 8B) was even faster than growth of WT *P. aeruginosa* (Fig. 4A). This confirms that PA14 deploys both contact-dependent and contact-independent weapons against *Synechocystis*; however, the strong negative impact of the H123^−^ mutation on PA14 growth in *Synechocystis* co-culture (Fig. 8) demonstrates the importance of the contact-dependent mechanism (T6SS) for PA14 growth in this condition. Taken together, our results indicate that contact-independent attack with pyocins and contact-dependent attack with T6SS are similarly effective at killing *Synechocystis* cells. However, *P. aeruginosa* cell proliferation is greatly impaired in the PA14 H123^−^ mutant lacking T6SS, whilst PA14 *prtN::tn*, which retains the T6SS but not pyocins, proliferates strongly in co-culture with *Synechocystis* (Fig. 8). This indicates that *P. aeruginosa* requires its T6SS to fully benefit from predation.

## Discussion

### *P. aeruginosa* employs its type VI secretion systems for predation on *Synechocystis*

Predation amongst bacteria is well established, with examples ranging from the intracellular parasitism of *Bdellovibrio* [38] to bacterial hunting by swarms of *Myxococcus xanthus* [39] *Pseudomonas aeruginosa* is highly motile and equipped with a formidable array of antibacterial weapons [12], often used to eliminate competitors [14]. Here, we show that *P. aeruginosa* can employ these weapons for direct predation, enabling proliferation in co-culture with a cyanobacterium in mineral medium, where organic compounds are only available as photosynthate from the cyanobacterium. Our results with *P. aeruginosa* mutants (Figs 6 and 7) and filtered *P. aeruginosa* medium (Fig. 6) indicate that *P. aeruginosa* can lyse *Synechocystis* cells by both contact-dependent and contact-independent mechanisms. Contact-independent mechanisms appear similarly effective for lysing *Synechocystis* cells but not for PA14 survival. Thus, a mutant lacking pyocins for contact-independent cell lysis thrives in *Synechocystis* co-culture, whereas the H123- mutant lacking T6SS survives but grows only very slowly (Fig. 8). T6SS are contact-dependent weapons that operate by direct injection of toxic proteins into target cells, leading to cell death [13, 40]. The H2-T6SS and H3-T6SS of *P. aeruginosa* have also been shown to be involved in metal iron acquisition with specific effectors that target iron, copper, and molybdate [41–44]. As the T6SS directly injects toxic bacterial-derived proteins into target cells, the advantage of this contact-dependent system is that target cell lysis releases cell contents into the milieu for immediate consumption by the adjacent attacking cell. By contrast, remote lysis by contact-independent systems releases cell contents which become diluted in the surrounding milieu. Our micrographs of *P. aeruginosa*:*Synechocystis* co-cultures support close contact between lysed *Synechocystis* cells and predating *P. aeruginosa* (Fig. 5)*,* but further work is required to understand the interaction dynamics. T6SSs are effective ‘disintegration weapons’ through their delivery of lytic toxins [45], and it is possible that this may serve as a particularly effective way to release prey cell contents for consumption. However, recent work has elegantly shown that killing by the T6SS using effector sets that result in slow lysis results in a graded release of nutrients allowing for more efficient use by the attacking organism [19], and this may be the case with predation on *Synechocystis.*

### Direct contact with foreign bacteria induces the formation of dense flocs of *Synechocystis*

Several studies have shown that *Synechocystis* cells assemble into flocs in appropriate conditions in pure planktonic cultures [4, 6, 7]. Here, we have shown that exposure to foreign bacteria induces the formation of dense *Synechocystis* flocs (Fig. 1) exhibiting enhanced extracellular polysaccharide production (Fig. 2). This dense flocculation is induced to similar extents by both the benign *E. coli* Top10 and the more aggressive *P. aeruginosa* PA14 (Fig. 1). Dense flocculation does not appear to be a specific response to attack, since it can be partially induced by heat-killed PA14 cells but not by exposure to filtered PA14 medium (Fig. 3), although this medium is effective in killing some *Synechocystis* cells (Fig. 6F). Our results suggest that dense flocculation is triggered by contact with the cell surfaces of foreign bacteria. It is likely that discrimination between ‘self’ and ‘non-self’ is involved, because flocculation is not a simple response to cell density: the addition of an equivalent number of extra *Synechocystis* cells does not trigger denser flocculation (Fig. 1). The method by which *Synechocystis* senses contact with foreign bacteria is unknown, but it is likely that T4P are involved. Although the requirement for floc formation of T4P [6, 7] and production of a sulfated EPS [4] is established, it is not clear whether T4P are required to trigger the response by forming initial cell–cell contacts, or by promoting EPS production, or both. T4P are involved in surface sensing in many bacteria [46], and *Synechocystis* is known to respond to surface contact with changes in gene expression [47]. In addition, *Synechocystis* encodes an extensive and diverse set of T4P minor pilins that may recognize multiple targets [48, 49]. As a working hypothesis, we suggest that *Synechocystis* pilus tips adhere to components found on foreign cell envelopes, and the resulting tension in the pilus triggers signal transduction via cyclic di-GMP [50] that leads to EPS production and dense floc formation [7, 9]. Previously*, P. taiwanensis* was shown to stabilize co-culture biofilms with *Synechocystis* [11] and another cyanobacterium [51]. The mechanism is unclear but was suggested to involve regulation of oxygen levels in the biofilms by *P. taiwanensis* [11]. In our experiments with planktonic cells and shaken culture wells with an airspace, O_2_ concentration is unlikely to be a significant variable, due to ready atmospheric equilibration. Nevertheless, the presence of heterotrophic bacteria can strongly induce *Synechocystis* cell aggregation. We suggest that direct contact sensing of *P. taiwanensis* in the biofilms may explain the previously observed stabilising effect.

### Flocculation in *Synechocystis* provides a defensive barrier against bacterial predation

Dense *Synechocystis* flocs are associated with high concentrations of extracellular polysaccharide, and microscopic examination of the edges of flocs suggest that they could provide an effective physical barrier to penetration of contact-dependent and independent mechanisms employed by bacteria such as *P. aeruginosa* (Figs 2 and 5A). Production of EPS capsules has been shown in another bacterium to be an effective defence against attack via T6SS [52]. Here, we show that *P. aeruginosa* proliferation in *Synechocystis* co-culture is greater with the nonflocculating *Synechocystis* Δ*hfq* mutant than with the WT (Figs 4 and 8), providing strong evidence that flocculation reduces predation. We cannot exclude other roles of flocculation such as flotation [4] or shading [8–10], and indeed, the roles of photosensors in promoting cell aggregation in *Synechocystis* [7] and other cyanobacteria [9, 10] suggest a role in modulating the light environment. However, encounters with predatory microbes are common in the environment. Amoebae and other protozoa are known to graze on cyanobacteria [53–55], and our laboratory experiments suggest that predation by heterotrophic bacteria may also be commonplace. We argue that a major function of cyanobacterial flocs is to provide protection from microbial predation.

### Alternative interpretations of the *Synechocystis*: *P. aeruginosa* interaction

It is clear from our data that *P. aeruginosa* can thrive in co-culture with *Synechocystis* by exploiting the photosynthate that *Synechocystis* produces, but does this indicate predation or some form of cross-feeding? The presence of *P. aeruginosa* is clearly deleterious for *Synechocystis* (Figs 4B, 6A and  7), but lysis of *Synechocystis* cells might be incidental to *P. aeruginosa* growth on some exported *Synechocystis* product. The strongest argument against such a scenario is that a *P. aeruginosa* mutant deficient in the T6SS is strongly impaired in growth in these conditions, in contrast to a mutant deficient in contact-independent weapons, which thrives (Fig. 8). This points to a direct link between the mode of *Synechocystis* cell lysis and the efficiency of utilisation of its photosynthate by *P. aeruginosa*.

*P. aeruginosa* grows better in co-culture with the nonflocculating Δ*hfq* mutant than with wild type *Synechocystis* (Figs 4A and 8). Conceptually, it would be logical for flocculation to confer defence against *P. aeruginosa* predation. Parallels can be drawn between the ability of swarming strains of *Proteus mirabilis* to invade opposing swarms of a rival strain dependent upon their T6SS [56]. As the T6SS is a contact-dependent weapon, and the length of the T6SS is limited by the width of the attacking cell, flocculation would provide a defence to prey killing as the inner cells cannot be directly targeted. However, an alternative explanation for our data could be that Δ*hfq* produces more photosynthate that can be exploited by *P. aeruginosa*. Indeed, the baseline growth of Δ*hfq* is substantially faster than wild type *Synechocystis* in our lab conditions (Figs 4B and 6A), likely driven by increased access to light and minerals. Our future experiments will focus on modulating the levels of EPS and the subsequent flocculation to probe this point further.

## Conclusions and future directions

Here we have shown that *P. aeruginosa* can lyse the cells of a cyanobacterium and can use the lysate for survival and proliferation in nutrient-poor conditions. Contact-dependent lysis of cyanobacterial cells by the type VI secretion system appears to allow faster *P. aeruginosa* proliferation than lysis by contact-independent methods, likely because contact-dependent lysis gives direct access to the released nutrients before they are diluted in the surrounding milieu. The cyanobacterium *Synechocystis* shows a behavioural response to contact with *P. aeruginosa* (and likely other foreign bacteria too): aggregation into dense flocs surrounded by extracellular polysaccharide which appears to serve as a defence against predation*. Pseudomonas aeruginosa* and *Synechocystis* have overlapping habitats on freshwater bodies and therefore these predation and defence mechanisms have likely ecological significance. Molecular details of the *Synechocystis*/*P. aeruginosa* interaction remain to be established, especially how *Synechocystis* recognizes the presence of foreign bacteria, leading to the stimulation of flocculation, and whether *P. aeruginosa* uses a specific T6SS for predation on cyanobacteria. Isotopic labelling experiments could provide more direct and specific evidence for the role of predation in nutrient acquisition by *P. aeruginosa*. Flocculation, sinking, and burial of phytoplankton are the crucial elements of the biological carbon pump [17, 57]. Heterotrophic bacteria are known to limit carbon burial by consuming organic matter [57], but if their presence stimulates flocculation of phytoplankton they could actually promote carbon burial too. Therefore, it will be important to establish whether the stimulation of flocculation by foreign bacteria also occurs in the most abundant marine cyanobacteria.

## Supplementary Material

SupplementaryDataB_wrag169

## Data Availability

All data generated or analysed during this study are included in this published article (and its supplementary information files).
