# Supplementary material for: Cyanobacterial flocculation as a defence against bacterial predation
Source: ISME J. 2026 Jun 27;20(1):wrag169. doi: 10.1093/ismejo/wrag169 (PMC13398385; doi:10.1093/ismejo/wrag169)
Supplement: SupplementaryDataB_wrag169 [file supplementarydatab_wrag169.docx]

**Cyanobacterial flocculation as a defence against bacterial predation**

Shylaja N. Mohandass, Alice C.Z. Collins, Fabian D. Conradi, Luke P. Allsopp and

Conrad W. Mullineaux

**Supplementary Material**

**Figure S1:** Contact between *Synechocystis* and *Pseudomonas aeruginosa* (PA14) cells.

**Table S1:** PCR primers used in generation of the PA H123- mutant.

**Table S2:** *P. aeruginosa* growth data from Fig 4A

**Table S3:** *Synechocystis* growth data from Fig 4B

**Table S4:** *Synechocystis* growth data from Fig 6A

**Table S5:** *P. aeruginosa* growth data from Fig 8A

**Table S6:** *P. aeruginosa* growth data from Fig 8B

**
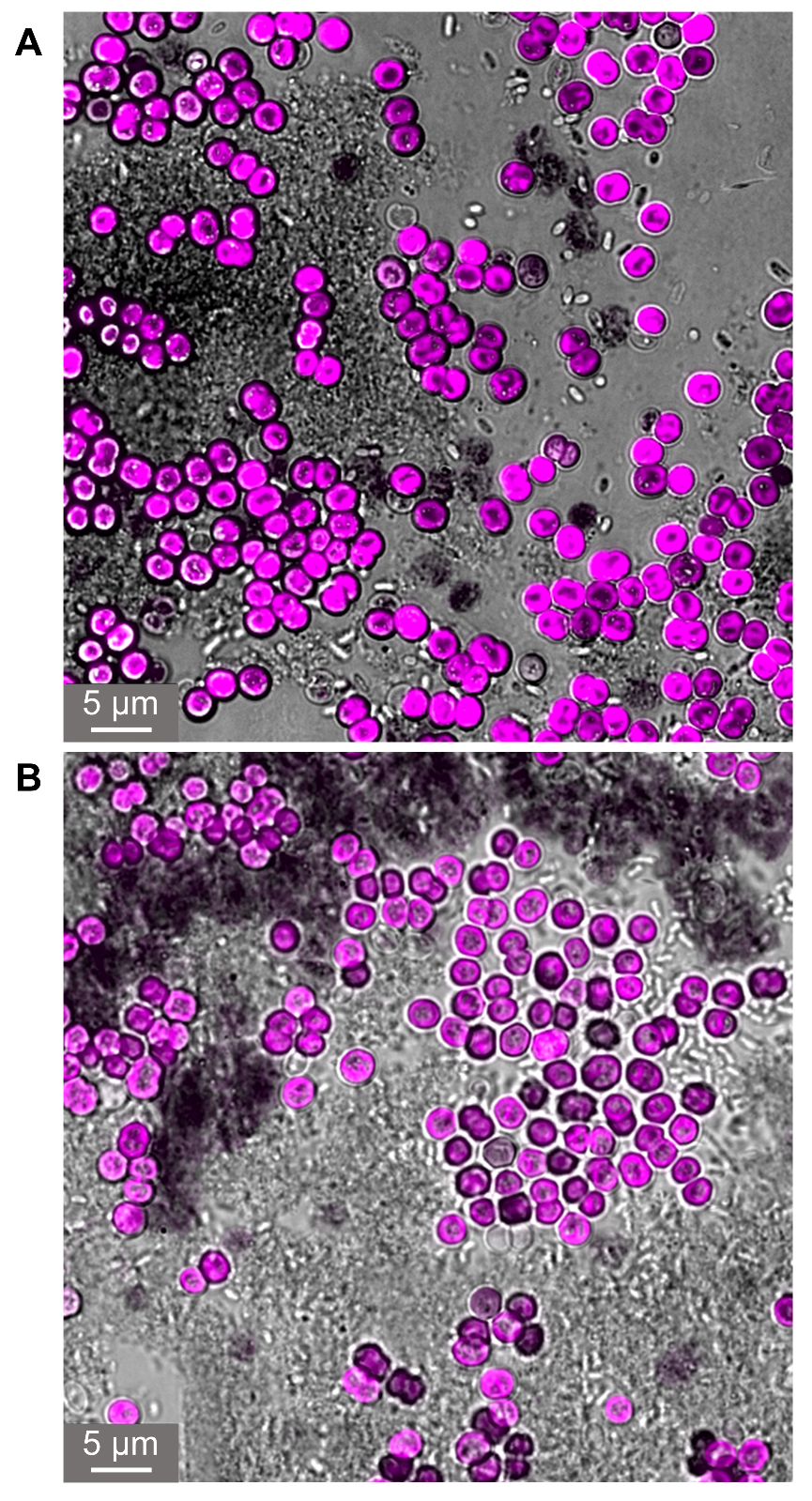
**

**Figure S1. Contact between *Synechocystis* and *Pseudomonas aeruginosa* (PA14) cells. A.** Wild type *Synechocystis*. **B.** *Synechocystis* Δ*hfq.* Confocal fluorescence /brightfield micrographs showing chlorophyll fluorescence in magenta and the brightfield channel in greyscale. *Synechocystis* cells are seen as round fluorescent bodies about 2.5 μm in diameter (with loss of fluorescence in lysed cells). *P. aeruginosa* cells appear as much smaller thin rods which may be bright or dark depending on their position relative to the focal plane. Greyscale settings have been adjusted in each image to optimise the visibility of *P. aeruginosa* cells.

**Table S1: PCR primers used in generation of the PA14 H123^-^ mutant**

| **Number** | **Brief description** | **Sequence (5ˊ-3ˊ)** |
| --- | --- | --- |
| P1 | ApaI.rsmA.F | ATATAGGGCCCCTTCAAGATCCTCGGGCCGATC |
| P2 | SmaI.rsmA.R | ATATACCCGGGGCAACTGTCGATCCTTCGTCCG |
| P3 | rsmA.Screen.F | GTCGAGTTCGCCGGCAAGTACAAC |
| P4 | rsmA.Screen.R | CTTTCGGTATGGCGCACTCAGG |
| P5 | Screen.UpKn.F | CATATCACAACGTGCGTGGA |
| P6 | Screen.RpKn.R | CCCTGGATTTCACTGATGAG |

**Table S2: *P. aeruginosa* growth data from Fig. 4A.**

|  | **PA14 + Δhfq** |  | **% increase in PA** |
| --- | --- | --- | --- |
|  |  |  |  |
|  | 0th hour | 48th hour |  |
| CFU count | 60000000 | 370000000 | 516.6667 |
|  | 50000000 | 370000000 | 640 |
|  | 30000000 | 420000000 | 1300 |
|  |  |  |  |
|  | **PA14** |  |  |
|  | 0th hour | 48th hour |  |
| CFU count | 28000000 | 47000000 | 67.85714 |
|  | 26000000 | 40000000 | 53.84615 |
|  | 26000000 | 46000000 | 76.92308 |
|  |  |  |  |
|  | **PA14 + 6803** |  |  |
|  | 0th hour | 48th hour |  |
| CFU count | 26000000 | 75000000 | 188.4615 |
|  | 26000000 | 80000000 | 207.6923 |
|  | 27000000 | 71000000 | 162.963 |

**Table S3: *Synechocystis* growth data from Fig. 4B.** Relative cell concentration estimated from chlorophyll concentration (in µM) in methanol extracts.

|  | 0th hour | 48th hour | **% increase in chlorophyll** |
| --- | --- | --- | --- |
|  | 2.49 | 5.908 | 137.2691 |
| wt | 2.59 | 5.88 | 127.027 |
|  | 2.646 | 6.58 | 148.6772 |
|  |  |  |  |
|  | 2.646 | 2.436 | -7.93651 |
| wt+PA14 | 2.618 | 2.38 | -9.09091 |
|  | 2.59 | 2.08 | -19.6911 |
|  |  |  |  |
| Δ*hfq* | 2.284 | 8.052 | 252.5394 |
|  | 2.17 | 8.82 | 306.4516 |
|  | 2.042 | 8.666 | 324.3879 |
|  |  |  |  |
| Δ*hfq*+PA14 | 2.306 | 3.528 | 52.99219 |
|  | 2.586 | 3.626 | 40.21655 |
|  | 2.492 | 3.57 | 43.25843 |

**Table S4: *Synechocystis* growth data from Fig. 6A.** Relative cell concentration estimated from chlorophyll concentration (in µM) in methanol extracts.

|  | 0th hour | 48th hour | % increase in chlorophyll |
| --- | --- | --- | --- |
|  | 2.674 | 2.282 | -14.65968586 |
| wt + PA14 H123^-^ | 2.73 | 2.268 | -16.92307692 |
|  | 2.772 | 2.226 | -19.6969697 |
|  |  |  |  |
|  | 2.59 | 2.632 | 1.621621622 |
| Δ*hfq* + PA14 H123^-^ | 2.59 | 2.604 | 0.540540541 |
|  | 2.646 | 2.548 | -3.703703704 |
|  |  |  |  |
|  | 2.856 | 2.394 | -16.17647059 |
| wt + *prtN::tn* | 2.912 | 2.394 | -17.78846154 |
|  | 2.842 | 2.422 | -14.77832512 |
|  |  |  |  |
|  | 2.744 | 2.506 | -8.673469388 |
| Δ*hfq* + *prtN::tn* | 2.716 | 2.548 | -6.18556701 |
|  | 2.786 | 2.506 | -10.05025126 |
|  |  |  |  |
|  | 2.926 | 11.522 | 293.7799043 |
| wt | 2.744 | 11.592 | 322.4489796 |
|  | 2.814 | 10.136 | 260.199005 |
|  |  |  |  |
|  | 2.7678 | 15.288 | 452.3520486 |
| Δ*hfq* | 2.884 | 15.316 | 431.0679612 |
|  | 2.968 | 15.148 | 410.3773585 |

**Table S5: *P. aeruginosa* growth data from Fig. 8A**

|  | **PA14 H123^-^** |  | **% of increase in PA** |
| --- | --- | --- | --- |
|  | 0th hour | 48th hour |  |
|  | 14000000 | 6000000 | -57.14285714 |
| CFU count | 26000000 | 6000000 | -76.92307692 |
|  | 15000000 | 5000000 | -66.66666667 |
|  |  |  |  |
|  | **H123^-^ + wt** |  |  |
|  | 0th hour | 48th hour |  |
|  | 22000000 | 30000000 | 36.36363636 |
| CFU count | 23000000 | 20000000 | -13.04347826 |
|  | 21000000 | 30000000 | 42.85714286 |
|  |  |  |  |
|  | **H123^-^ + Δ*hfq*** |  |  |
|  | 0th hour | 48th hour |  |
|  | 21000000 | 31000000 | 47.61904762 |
| CFU count | 19000000 | 26000000 | 36.84210526 |
|  | 21000000 | 23000000 | 9.523809524 |

**Table S6: *P. aeruginosa* growth data from Fig. 8B**

|  | **PA14 *prtN::tn*** |  | **% of increase in PA** |
| --- | --- | --- | --- |
|  | 0th hour | 48th hour |  |
|  | 10000000 | 6000000 | -40 |
| CFU count | 30000000 | 6000000 | -80 |
|  | 18000000 | 5000000 | -72.22222222 |
|  |  |  |  |
|  | ***prtN::tn* + wt** |  |  |
|  | 0th hour | 48th hour |  |
|  | 20000000 | 90000000 | 350 |
| CFU count | 10000000 | 50000000 | 400 |
|  | 10000000 | 90000000 | 800 |
|  |  |  |  |
|  | ***prtN::tn* + Δ*hfq*** |  |  |
|  | 0th hour | 48th hour |  |
|  | 100000000 | 4500000000 | 4400 |
| CFU count | 100000000 | 4100000000 | 4000 |
|  | 100000000 | 3800000000 | 3700 |
